# Health economics analysis of restrictive school smartphone policies in secondary schools in England (SMART Schools)

**DOI:** 10.1136/bmjment-2025-301892

**Published:** 2026-02-10

**Authors:** Samuel J Perry, Victoria A Goodyear, Miranda Pallan, Peymane Adab, Sally Fenton, Maria Michail, Paul Patterson, Amie Randhawa, Alice J Sitch, Matthew Wade, Hareth Al-Janabi

**Affiliations:** 1 Health Economics Unit, School of Health Sciences, College of Medicine and Health, University of Birmingham, Birmingham, UK; 2 School of Sport, Exercise and Rehabilitation Sciences, College of Life and Environmental Sciences, University of Birmingham, Birmingham, UK; 3 Institute for Mental Health, University of Birmingham, Birmingham, UK; 4 Department of Applied Health Sciences, School of Health Sciences, College of Medicine and Health, University of Birmingham, Birmingham, UK; 5 Birmingham Biomedical Research Centre, National Institute for Health and Care Research, Birmingham, UK; 6 Centre for Youth Mental Health, University of Melbourne, Parkville, Victoria, Australia; 7 School of Psychology, University of Birmingham, Birmingham, UK; 8 Birmingham and Solihull Mental Health NHS Foundation Trust (BSMHFT), Birmingham, UK; 9 ukactive Research Institute, London, UK

**Keywords:** Mental Health

## Abstract

**Background:**

Many countries have introduced restrictive smartphone policies in schools, aiming to improve adolescent health and educational outcomes. However, whether these policies represent value for money to schools remains unclear.

**Objective:**

The aim of this study was to estimate the costs and quality of life and mental well-being outcomes associated with restrictive and permissive phone policies in secondary schools in England, and conduct an economic evaluation to determine whether restrictive phone policies are cost effective for schools.

**Methods:**

A cost–utility analysis was conducted as part of a cross-sectional study on school smartphone policies in England (SMART Schools), comparing schools with restrictive (recreational phone use not permitted) and permissive (recreational phone use permitted) policies. Outcomes were valued using quality-adjusted life years (QALYs) and mental well-being adjusted life years (MWALYs). Costs were estimated from the school’s perspective and comprised staff time spent on policy implementation. Mixed effects and linear regression models were used to estimate incremental differences in outcomes and per pupil costs.

**Findings:**

815 pupils (aged 12–15) from 20 schools (13 restrictive, 7 permissive) were included in a complete case analysis. Differences between restrictive and permissive schools in incremental QALYs (0.009, 95% CI −0.014 to 0.032) and MWALYs (−0.004, 95% CI −0.044 to 0.036) were minimal. Time implementing and enforcing policies was equivalent to 3.1 FTE staff in restrictive and 3.3 FTE staff in permissive schools. The incremental difference in per pupil school year cost was −£94 (95% CI −£229 to £41). The cost-effectiveness acceptability curve indicated a 90% probability of restrictive policies being cost effective at a threshold of £20 000 and £30 000 per QALY.

**Conclusions:**

Restrictive school policies were associated with minimal differences in quality of life or mental well-being of pupils. However, they may offer some cost savings to schools by reducing staff time spent managing phone-related activities.

**Clinical implications:**

School policies and practices require development to address the significant amount of time teachers spend managing phone use.

**WHAT IS ALREADY KNOWN ON THIS TOPIC**

- Previous UK studies, including SCAMP and SMART Schools, have explored the impact of school smartphone policies on adolescent outcomes. Although restrictive policies may reduce device use, evidence suggests they do not significantly improve adolescent mental health and well-being, physical activity and sleep or educational attainment, and classroom behaviour. Published, peer-reviewed studies reporting economic evaluations of school phone policies are needed.

**WHAT THIS STUDY ADDS**

- This study provides an economic evaluation of school smartphone policies, comparing costs and pupil outcomes between schools with restrictive and permissive smartphone policies. The findings suggest that schools are spending a significant amount of staff time each week administering smartphone policies and managing phone-related behaviours. Restrictive policies may be cost-saving for schools, primarily by reducing staff time spent on phone-related monitoring and administration, but do not have a substantial impact on pupils’ quality of life or mental well-being.

**HOW THIS STUDY MIGHT AFFECT RESEARCH, PRACTICE OR POLICY**

- This study provides further evidence that there are unlikely to be differences in pupils’ mental health and well-being outcomes in adolescents attending schools with a restrictive or permissive smartphone policy. Restrictive phone policies could offer small economic benefits to schools by reducing the amount of time school staff spend managing pupil phone-related behaviours. Overall, the findings highlight the need for development of current school phone policy and practices to reduce school staff time spent managing adolescent phone use, potentially freeing up resources for more beneficial educational and well-being activities.

## Background

 Mental health problems are prevalent during adolescence (ages 10–19).^1^ In the UK, an estimated one in five adolescents aged 11–16 has a probable mental health disorder (mostly anxiety and depression).^2^ Concurrently, there has been a rapid rise in the ownership of smartphones and the use of social media. By age 12, most adolescents own a phone and use social media.^3^ Adolescents reportedly spend between 4 and 6 hours per day on their phones and 2–4 hours per day on social media.^4 5^ Although evidence about the overall impact of phones and social media on adolescents is not clear cut,^6^ increased time spent on phones and social media tends to be associated with worsened mental health outcomes.^5^

There is a growing international trend to restrict the use of phones in schools to mitigate the harmful effects of phones and social media.^7^ A number of countries (in Europe and regions of the USA and Canada) have introduced laws, policies or guidance for schools to ‘ban’ or heavily restrict the use of phones.^8^ Restrictions vary, with some schools not allowing any devices on school premises, others requiring students to put phones away in a locked bag (eg, a pouch), while others allow students to have their phones in their bags or pockets but they are not allowed to use them.^6 7^ Some evidence exists that restrictive school phone policies lower the time adolescents spend on their phones and social media during the school day.^5 9^ Some studies have shown benefits when phone use in school is restricted for students’ academic performance, particularly among the lowest achieving students.^10^ However, a recent evaluation of school smartphone policies in England, conducted by the authors of this study, reported that restricted smartphone use in schools was not associated with benefits to adolescent mental health and well-being, and other health and educational outcomes.^5^

Before the introduction of legislation and guidance, many schools devised their own policies to restrict phone use.^7 8^ The implementation of such policies was based on assumptions that restricting phone use during the school day would reduce bullying, limit distractions to learning and minimise safeguarding-related concerns.^7 8 10^ Teachers, pupils and parents tend to be supportive of restrictive school phone policies^7^ and have cited benefits relating to behaviour in schools after their introduction.^8^ Therefore, there may be other, broader benefits to restrictive school phone policies that have yet to be established.^5 10^

Health economics analysis can present decision-makers with an assessment of both the costs and health outcomes associated with a new intervention. From a school’s perspective, the main cost to consider is the opportunity cost of staff time spent implementing a phone policy and/or managing the consequences of permitted phone use during the school day. For example, responding to phone-related incidents (eg, bullying or safeguarding) that happen during the school day may divert staff time away from other activities. Economic analysis may help school decision-makers understand the costs or cost savings that phone policies may incur, alongside the health and quality-of-life benefits (or harms) they may provide for pupils.

The cost effectiveness of school phone policies has not been previously assessed. Economic analyses of other school-based interventions have been conducted, including whole-school interventions targeting social and emotional learning, mindfulness and resilience, and anti-bullying.^11^,^14^ Often, these studies have found small to negligible effects on health outcomes, but have reported interventions to be cost effective at conventional willingness-to-pay thresholds (ie, the maximum society is willing to spend for a given health benefit) due to their low implementation costs.^11^,^14^

## Objective

The objective of this study was to conduct an economic evaluation to determine whether restrictive phone policies in schools offer value for money to schools in terms of pupils’ quality of life and mental well-being outcomes.

## Methods

### Study design and participants

The economic evaluation was nested in the SMART Schools study, a natural experimental observational study designed to evaluate the impact of school phone policies on mental well-being.^5^ Full details of the study are reported in the protocol.^15^ This economic evaluation is reported in accordance with the Consolidated Health Economic Evaluation Reporting Standard (CHEERS) 2022 guidelines (online supplemental materials, pp 19–21).

The recruitment process is outlined in online supplemental figure S1. The sampling frame included 1341 state-funded mainstream secondary schools (pupils aged 11–18) within 100 miles of the recruiting centre. Specialist provision schools, pupil referral units and independent schools were excluded. Schools with different phone policies for different year groups and/or with inaccessible smartphone policies were also excluded.

Schools within the sampling frame were classified as either having a ‘restrictive’ (intervention) or ‘permissive’ (comparator) smartphone policy (online supplemental table S1), which were informed by patient and public involvement (PPI) activities, and school website and policy analysis.^7 15^ In schools with restrictive policies, phones were not allowed to be used during the school day for recreational purposes and were required to be turned off and inside bags, stored in lockers, kept in a pouch, handed into the school reception or not allowed onto the premises. In permissive schools, phones were allowed to be used at any time or at certain times (eg, breaks/lunch), and/or in certain zones (eg, outside).

To improve comparability between schools with restrictive and permissive policies, stratified sampling based on propensity scores was employed.^5 15 16^ Routine data obtained from the Department for Education on school characteristics were used, including school type, urban versus rural, single sex schools, Income Deprivation Affecting Children Index (IDACI), inclusion of a sixth form, selective admissions policy, religious affiliation and the proportion of pupils with the following characteristics: male, from minority ethnic groups, English as an additional language and special educational needs (online supplemental table S2). Propensity scores were calculated by regressing policy type (permissive or restrictive) on these characteristics. Schools were then grouped into three categories based on propensity score terciles, with each category further divided by school phone policy type, resulting in six distinct sampling groups. Schools in each group were randomly ordered and invited sequentially to participate. The final sample consisted of 20 schools with restrictive policies and 10 schools with permissive policies.

Data were collected between November 2022 and November 2023. Self-administered online surveys were completed by pupils (year 8 (aged 12–13) and year 10 (aged 14–15)), teachers (two from each school, the year 8 and year 10 form tutors or equivalent), and a member of the school senior leadership team (SLT) with responsibility for the school’s smartphone policy (eg, school behaviour, mental health or safeguarding lead). The survey questions were co-produced and piloted as part of the study PPI activities.

### Costs

Costs were generated from the perspective of the school and estimated for the duration of the school year (39 weeks), and as such, discounting was not required.^17^ All costs are reported in GBP in 2022/23 prices.

The primary area of resource use was staff time for the day-to-day implementation of the school’s phone policy. Time spent developing the policy was not considered because in all cases this took place in previous school years. Time use was considered for the following: academy trust board; headteacher; SLT; school governors; teachers; teaching assistants; safeguard/welfare support staff; school reception staff; building managers/caretakers; cleaners; other.

Resource use data were collected via two methods. First, a survey administered to one member of the school’s SLT team responsible for the school’s phone policy, asking them to report the number of hours spent on day-to-day policy implementation (with examples of tasks provided, such as monitoring behaviour, recording incidents, duty roles, detentions, communicating with parents, talking to pupils, managing phones, supporting other school staff, providing training to other staff) in a typical week by staff members in the aforementioned groups (online supplementary materials, pp 12–13). Second, a survey administered to one or two teaching staff members per school asking them to describe their role and give a detailed breakdown of the time they spent applying sanctions and monitoring behaviour related to the school’s phone policy (online supplemental materials, p 14). For costing purposes, the SLT survey data were used as these provided an overall estimate of the time spent by staff within the school administering their phone policy.

Local authority level mean salaries (online supplemental table S3) were applied to staff time estimates for the following: headteacher, SLT and teaching staff (teachers, teaching assistants and safeguarding or welfare support staff).^18^ For non-teaching staff, estimates were obtained from the mid-point (spine 7) of the National Joint Council Local Government Pay Scales, equivalent to £11.53 per hour.^19^ The academy trust board and school governors were omitted as no schools reported their involvement in the day-to-day implementation of the phone policy. The reported total typical weekly hours were then multiplied by 39 weeks to provide a school year estimate for the total hours. The total school year cost for the four staff groups was summed to provide a total school year cost for each school for their phone policy implementation. This was divided by the roll size of the school to provide a per pupil school year cost and matched to the pupil data by school.

### Outcomes

This study used two generic health-related outcomes commonly used in economic evaluations: quality-adjusted life years (QALYs), which combines quality and length of life into a single metric, and mental well-being adjusted life years (MWALYs), which similarly combines mental well-being status and length of life. The key difference between the measures is MWALYs’ sole focus on mental health and well-being, making the metric potentially more sensitive to changes in these aspects compared with QALYs. Other outcomes relevant to schools, including phone and social media use, educational outcomes and behaviour have been reported elsewhere.^5^ Pupils completed the Child Health Utility (CHU9D) at baseline and the Warwick-Edinburgh Mental well-being Scale (WEMWBS) at two time points approximately 4–8 weeks apart (online supplemental materials, pp 15–18).^20 21^

The CHU9D is a paediatric quality-of-life instrument validated for use with adolescents up to 17 years old.^20^ The WEMWBS is a 14-item instrument measuring positive aspects of mental health.^21^ Since a tariff is only available for the ‘short’ version of the WEMWBS (SWEMWBS) consisting of a subset of seven items, this was used to calculate the utility values to derive MWALYs for the analysis.^22^

QALYs were calculated using the utility value obtained from the single measurement of the CHU9D by extrapolating this value across the 39-week school year. For the calculation of MWALYs, the utility value was either calculated as the mean for those pupils who responded to the SWEMWBS at both time points or the single value for those with one missing response. These values were then extrapolated in the same manner as in the calculation of QALYs.

### Statistical analyses

The base-case analysis considered complete cases with both costs (at the school level) and outcomes (at the pupil level) available, and schools that returned erroneous or implausible estimates for staff time removed (online supplemental figure S2). The cost–utility analysis was conducted using adjusted models to obtain estimates for differences in outcomes and costs between restrictive and permissive schools through multivariate regression methods detailed below. All analyses were conducted in Stata 18.

Mixed-effects linear regression models were estimated for QALYs and MWALYs. School year (year 8 or year 10) and school were included as random effects to reflect the clustered nature of the data, and pupil-level and school-level control variables were included as fixed effects. Pupil-level variables were as follows: sex (male, female, prefer not to say); ethnicity (Asian/Asian British, black/African/Caribbean/black British, mixed/multiple ethnic groups, white, other ethnic group/prefer not to say); and season of response (summer (April–August) or otherwise). School-level variables were as follows: IDACI (where a higher decile indicates greater levels of deprivation); roll size (ie, number of pupils enrolled in the school); religious affiliation (secular or religious); admissions policy (selective or non-selective); school sex (co-educational or single sex); and school percentage of pupils with special needs education, English as an additional language and free school meals.

A multivariate linear model was estimated for per pupil school year cost, adjusting for the same school-level variables described above. Pupil-level characteristics were not controlled for because costs were derived from school-level data and then matched to pupils. Robust standard errors clustered at the school level were estimated.

Results are presented by the predicted mean QALYs, MWALYs and per pupil school-year costs for restrictive and permissive schools, and the incremental difference in the values obtained from the estimated coefficients. If appropriate, incremental cost-effectiveness ratios (ICERs) were obtained by dividing the estimated difference in cost per pupil between restrictive and permissive schools by the estimated difference in QALYs or MWALYs between the restrictive and permissive schools.

To address sample uncertainty around the estimates, a non-parametric bootstrap method (1000 iterations) was performed.^23^ Results were plotted on cost-effectiveness planes (CEPs), and a cost-effectiveness acceptability curve (CEAC) using a net monetary benefit approach.^24 25^ The CEAC allows for an estimation of the probability of cost effectiveness at increasing willingness-to-pay thresholds for health outcome gains.

To address missing data, a sensitivity analysis was conducted using multiple imputation via chained equations to impute missing pupil outcomes and missing time use information in the SLT data (which were subsequently then used to generate the cost variable).^26^ Multiple imputation via chained equations was performed using predictive mean matching with five nearest neighbours. Outcome models used the same pupil-level and school-level variables as the base-case regression models; the cost model used the school-level predictors only. Imputed costs were then matched to imputed pupil outcomes data. Given the rate of missingness in our data, 30 imputed datasets were generated^27^; estimates for the incremental QALYs, MWALYs and costs per pupil per school year were combined using Rubin’s rules.^28^

Deterministic sensitivity analyses were also conducted using alternative salary assumptions (online supplemental table S3). Finally, schools which returned implausible but not erroneous staff time estimates were included in a sensitivity analysis, and the base-case models were re-estimated to explore the impact of omitting outliers from the analysis.

## Results

Data were collected for 1227 pupils (820 from restrictive and 407 from permissive schools; 594 from year 8 and 633 from year 10 classes), 54 teachers (35 from restrictive and 19 from permissive schools) and 30 SLT staff (20 from restrictive and 10 from permissive schools).

Complete outcomes data were available for 1196 pupils (97.5%; 803 (97.9%) in restrictive schools, 393 (96.6%) in permissive schools) and complete time-use data were available from 20 schools (66.7%), yielding 815 pupil observations (online supplemental table S4 and figure S2). Online supplemental tables S5-S7 indicate missingness in school time-use data did not vary by school phone policy group (online supplemental table S5) or school characteristics (online supplemental table S6), while pupil health outcomes did not significantly differ between schools with and without missing time use data (online supplemental table S7). These results suggest that our data were likely to be missing at random.

### Descriptive analysis

Table 1 provides descriptive statistics for the complete case sample for pupils, school characteristics and school phone policy time use data, stratified by phone policy. Pupil characteristics were broadly similar across schools. For school-level characteristics, roll size was similar in both school groups (1025 pupils in restrictive schools and 1056 pupils in permissive schools). Headteacher and SLT time spent on phone policy implementation was similar across restrictive and permissive schools. However, restrictive schools’ teaching staff spent approximately 19 hours per week less than in permissive schools (75 hours in restrictive, 94 hours in permissive schools), but more burden fell on non-teaching staff (12 hours in restrictive, 0.5 hours in permissive schools).

**Table 1:** Descriptive statistics for base-case sample

Characteristics	Restrictive schools (n=13; pupils n=535*)	Permissive schools (n=7; pupils n=295*)	Statistical comparison test result (P value)
**Pupil level characteristics**			
Sex			0.57
Male	240 (45.5)	139 (47.4)	0.59
Female	284 (53.8)	150 (51.2)	0.48
Prefer not to say	4 (0.08)	4 (1.4)	0.40
Ethnicity			<0.01
White	405 (76.0)	190 (64.4)	<0.01
Asian, Asian British	59 (11.1)	65 (22.0)	<0.01
Black, African, Caribbean, black British	19 (3.6)	6 (2.0)	0.22
Mixed/multiple ethnic groups	29 (5.4)	23 (7.8)	0.18
Other ethnic group, prefer not to say	21 (3.9)	11 (3.7)	0.88
Season of response			
Summer	184 (34.5)	205 (69.5)	<0.01
Non-summer	349 (65.5)	90 (30.5)	
Year group			
Year 8	259 (48.6)	145 (49.2)	0.88
Year 10	274 (51.4)	150 (50.8)	
**School level characteristics**			
Roll size, mean (SD)	1025 (392)	1056 (280)	0.86
Religious affiliation: secular	13 (100.0)	5 (71.4)	0.04
Admissions policy: non-selective	12 (92.3)	4 (57.1)	0.06
School sex: single sex	1 (7.7)	2 (28.6)	0.21
Percentage special education needs, mean (SD)	12.7 (6.0)	9.1 (7.6)	0.26
Percentage English as an additional language, mean (SD)	11.9 (14.3)	15.4 (12.3)	0.65
Percentage free school meals, mean (SD)	17.6 (8.3)	13.7 (11.8)	0.40
Income Deprivation Affecting Children Index, mean (SD)	5.4 (2.1)	5.1 (3.0)	0.83
**School phone policy time**			
Weekly time (hours), mean (SD)			
Headteacher	1.05 (1.39)	0.17 (0.37)	0.12
Senior leadership team	13.66 (17.45)	13.29 (10.95)	0.96
Teaching staff	75.32 (63.49)	93.71 (88.59)	0.60
Non-teaching staff	11.62 (24.01)	0.46 (1.12)	0.24
Total	101.64 (96.28)	107.63 (99.30)	0.90
School year (hours), mean (SD)			
Total time	3963 (3755)	4198 (3872)	0.90
Per pupil time	3.907 (2.883)	4.761 (5.182)	0.64

Data are numbers (%) unless indicated otherwise.

* Complete data on pupil-level characteristics were available for 830 pupils; however, for the complete case sample used in the regression models, 14 pupils had missing outcome data. Statistical tests for differences between restrictive and permissive schools included χ2 tests for categorical variables and t-tests for each variable within a categorical variable, binary variables and continuous variables.

Overall, the total time spent managing smartphones in restrictive schools (102 hours) was approximately 6 hours per week lower than permissive schools (108 hours).

Across a 39-week school year, this is equivalent to 3963 hours in restrictive schools and 4198 hours in permissive schools. Assuming the school year full-time equivalent (FTE) hours for staff are 1265 hours per school year,^17^ this corresponds to approximately 3.1 FTE staff in restrictive schools and 3.3 FTE staff in permissive schools.

Consistent with the SLT-reported estimates, the self-reported weekly time spent by staff (online supplemental table S8) was lower in restrictive schools (7.3 hours) compared with permissive schools (9.1 hours), although this difference was not statistically significant (p=0.67). Teachers in restrictive schools reported spending the most time on ‘Discussion with parents (by phone or in school)’ (1.5 hours), ‘Sending text, letter or email to parents’ (1.0 hour) and ‘1:1 meetings with pupils’ (1.0 hour). Teachers in permissive schools reported spending the most time on ‘Recording incidents (eg, in planner or on school system)’ (2.7 hours), ‘1:1 meetings with pupils’ (0.9 hour), and ‘Discussion with parents (by phone or in school)’ (0.8 hours).

#### Cost–utility analysis

Table 2 presents the base-case cost–utility analysis results (see online supplemental table S12) for full regression results. Mean school-year costs per pupil were estimated to be −£94 lower (95% CI −£229 to £41) in restrictive schools. Estimated mean QALYs were marginally higher (0.009; 95% CI −0.014 to 0.032) in restrictive schools. As such, restrictive phone policies could be considered ‘dominant’ since they were associated with QALY gain (although marginal) at a lower cost.

**Table 2:** Cost–utility analysis—base-case results

Cost and outcomes	Restrictive schools (n=524)	Permissive schools (n=291)	Difference*	ICER
School year total cost per pupil (£)	104.71 (36.79 to 172.63)	198.76 (109.12 to 288.40)	−94.05 (−229.25 to 41.14)	—
QALYs	0.607 (0.597 to 0.618)	0.598 (0.581 to 0.614)	0.009 (−0.014 to 0.032)	Dominant
MWALYs	0.516 (0.493 to 0.549)	0.521 (0.493 to 0.549)	−0.004 (−0.044 to 0.036)	£23 513/QALY lost

Data are presented as mean (95% CI).

* See online supplemental table S12 for full regression results for the models estimating the difference in school year total cost per pupil, QALYs and MWALYs.

ICER, incremental cost-effectiveness ratio; MWALY, mental well-being adjusted life year; QALY, quality-adjusted life year.

For MWALYs, a marginal incremental loss of 0.004 (95% CI −0.044 to 0.036) was estimated for pupils in restrictive schools compared with permissive schools. Here, the point estimates suggest that on average, restrictive phone policies were cost-saving at the expense of a very small loss in pupils’ mental well-being (£23 513 saved per QALY lost).

**Figure 1:**
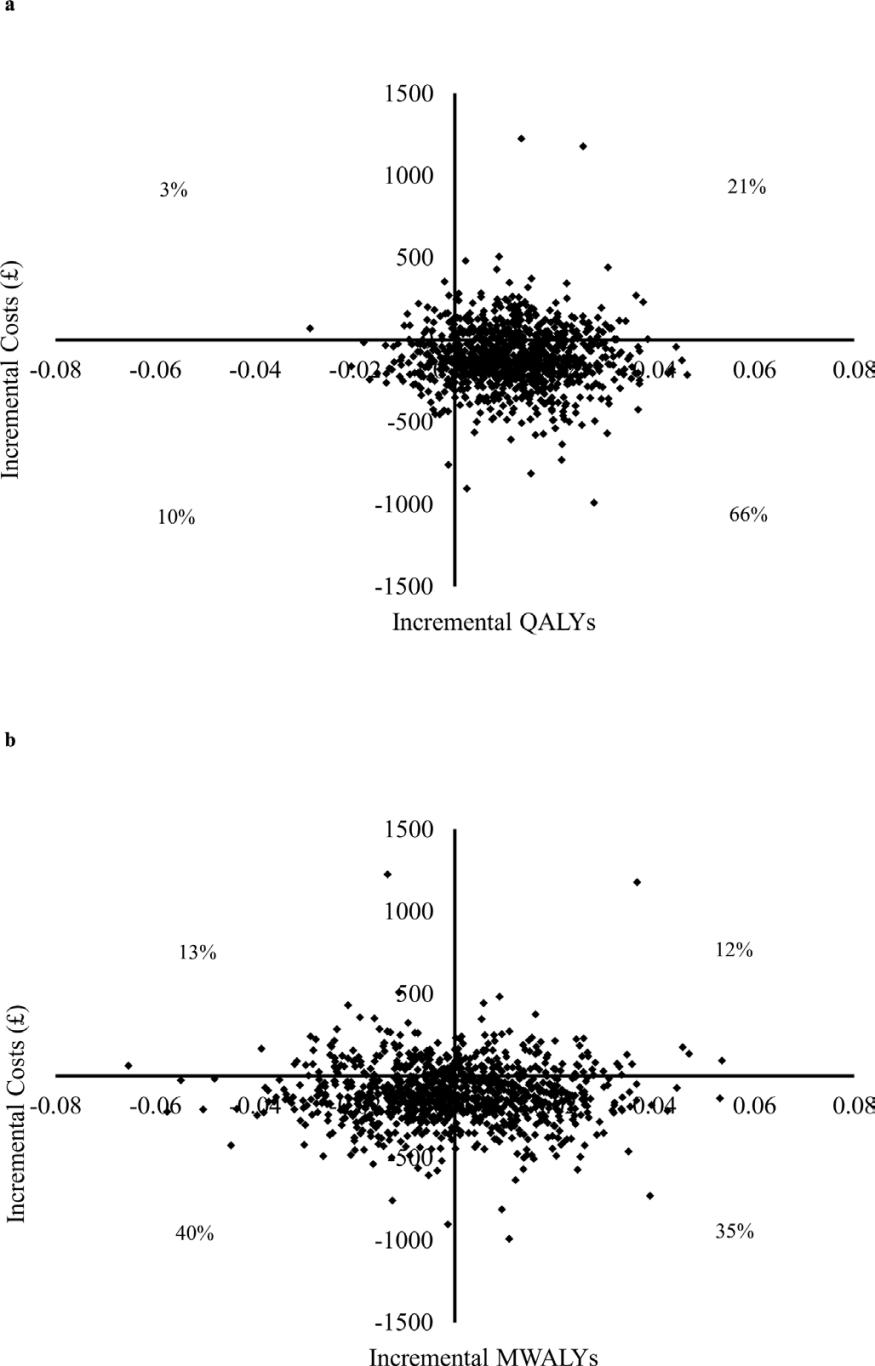
(a) Cost-effectiveness plane QALYs (quality-adjusted life years). (b) Cost-effectiveness plane MWALYs (mental well-being adjusted life years).

Bootstrapped regression results indicated that 76% of the 1000 replications estimated restrictive policies to be cost-saving; that is, costs per pupil were less for restrictive phone policies compared with permissive phone policies. However, there were differences between where the bootstrapped estimates lay dependent on the outcome considered. For QALYs (figure 1, upper panel), 66% lie in the south-east quadrant of the CEP; that is, where restrictive phone policy dominates permissive phone policy because it incurs less cost and leads to a QALY gain. However, there is greater uncertainty when considering MWALYs as the outcome (figure 1, lower panel) with 36% of bootstrapped estimates lying in the south-east quadrant, reflecting restrictive policies being dominant over permissive policies.

**Figure 2:**
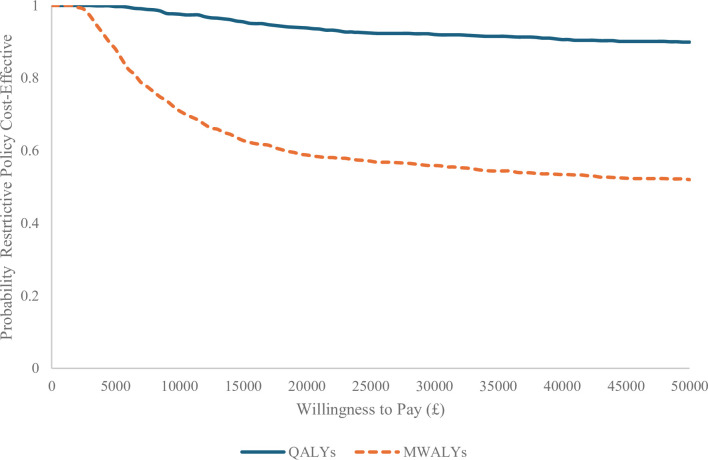
Cost-effectiveness acceptability curve. MWALY, mental well-being adjusted life year; QALY, quality-adjusted life year.

To contextualise the likelihood of restrictive phone policies being considered cost-effective, figure 2 provides CEACs with QALYs (solid line) and MWALYs (hatched line) as the outcomes. Note that where incremental costs were estimated to be negative, the net monetary benefit approach adds this value to monetary value of the incremental health gain or loss. At the lower and upper bounds of the NICE (National Institute for Health and Care Excellence) recommended thresholds of £20 000–£30 000 per QALY,^29^ restrictive policies had an 89.6% and 90.1% probability of being cost effective. Equivalent probabilities were lower for MWALYs (59.4% and 55.7%). Collectively, these results suggest a high certainty of restrictive phone policies being cost effective when considering QALYs. However, there is greater uncertainty in whether they would be cost effective if considering MWALYs, although there are also currently no accepted willingness-to-pay thresholds for MWALYs.

### Sensitivity analysis

The results from the multiple imputation analysis using data for 1227 pupils and all 30 schools (online supplemental table S9) and the deterministic sensitivity analyses including estimation using alternative salary estimates (online supplemental table S10) and the inclusion of two schools that returned implausible staff time estimates (online supplemental table S11) provide the same conclusion as the base-case analysis; restrictive phone policies appear to negligibly impact quality of life and well-being compared with permissive phone policies, but may lead to cost savings in terms of reduced teacher time allocated to policy implementation, although there is uncertainty around this estimate reflected by the 95% CIs. It should be noted that the direction of the estimated coefficient for restrictive schools in QALY model changes to negative in the multiple imputation analysis; however, this estimate remains negligible in magnitude and therefore does not alter the above conclusion.

## Discussion

This study found there were negligible differences in quality of life and mental well-being outcomes between pupils attending schools that permitted phone use compared with pupils attending schools that restricted phone use. The point estimates suggest minimally higher QALYs (0.009; 95% CI −0.014 to 0.032) but minimally lower MWALYs (−0.004; 95% CI −0.044 to 0.036) for pupils in restrictive schools. However, as these are two different measures with high uncertainty around the point estimates, it is expected they will not be perfectly correlated. When accounting for the estimated costs of the differing policies, in the case of QALYs, restrictive policies were likely to be cost effective.

Cost analyses indicated school staff spend a large proportion of their time managing phone use in schools, equivalent to more than 3 FTE staff across the school year, regardless of phone policy type. After adjustment for pupil-level and school-level characteristics, we found restrictive phone policies were potentially cost-saving to schools compared with permissive policies. Restrictive phone policies may therefore provide some economic benefits by reducing the amount of time school staff spend managing phone-related behavioural issues. The study findings indicate that, overall, current school phone policies and practices require development to reduce the amount of time school staff spend managing adolescent phone use during the school day, which may be diverting staff time away from other more beneficial educational and well-being activities.

The findings reinforce earlier analyses we reported from the SMART Schools study, which found no difference in mental health and other associated health and educational outcomes in adolescents attending schools with permissive or restrictive phone policies.^5^ The findings associated with the economic evaluation in the present study align with those from a variety of different school-based interventions, which tend to find minimal impacts on health outcomes among school-age children, but owing to low per pupil implementation costs, may often still represent value for money relative to conventional cost-effectiveness thresholds.^11^,^14^

We observed modestly higher staff time costs per pupil in the implementation of permissive phone policies compared with restrictive phone policies, although there was uncertainty around this estimate. This was unlikely to be significantly driven by the types of staff members involved in policy implementation, with different staff categories contributing to a similar share of total staff time in restrictive and permissive schools. However, as might be expected, staff in restrictive schools appear to spend less time on monitoring phone-related activities and administration duties (eg, recording incidents, providing staff with information and training), but more time applying behavioural sanctions for breaches of phone policy (eg, detentions and parent communication).

This study assessed value for money of school policies that restrict phone use in schools. We analysed the implementation of school policies in a carefully selected and nationally representative sample of 20 secondary schools and health outcomes in a relatively large cohort, with analysis adjusted for both school-level and pupil-level characteristics. Nevertheless, there are some limitations. This was a cross-sectional observational study exploiting existing variation in school phone policies. As such, we did not have data on costs and outcomes before and after the implementation of a more restrictive phone policy, and therefore could not analyse the changes in outcomes and resource use due to policy introduction. Furthermore, the collection of the outcome data for pupils was limited to one (CHU9D) or two (WEMWBS) assessment periods, with the timing varying across schools. As such, the derived QALY and MWALY estimates reflect snapshots of pupils’ quality of life and mental well-being, which were assumed to approximate the school year. While this challenges the internal validity of the study, we included seasonal fixed effects in the regression models to partially account for timing differences in collection of pupil data across schools. Additionally, given this time frame for data collection, we were unable to assess longer term costs and benefits of phone policies beyond a single school year. These may differ in the long run, however it is unclear what direction this would go in.

There were also some differences in the permissive and restrictive samples of schools, including a greater proportion of white pupils and a lower proportion of Asian or Asian British pupils in restrictive schools, and a greater proportion of secular schools in the restrictive schools group. However, this broadly reflected the differences in the permissive and restrictive schools in the sampling frame. Both pupil and school characteristics were also controlled for in the analysis, and therefore, despite some limitations, the results are likely to be applicable across England.

Although pupil outcome missingness was minimal, only 77% of schools provided complete data required for costing, and a further three returned implausible staff time estimates, reducing our base-case sample to 66% of schools from the SMART Schools study. While missingness did not significantly vary by phone policy group, school characteristics or pupil outcomes, it nevertheless reduced the sample used for our base-case analysis. Given these data were found to likely be missing at random, we could validly address this missingness using multiple imputation analysis. We found this did not significantly alter our conclusions that restrictive phone policies have a negligible impact on pupil outcomes, but are likely to be less costly to implement than permissive phone policies.

Our costing strategy relied on one senior staff member reporting the average time spent by different types of staff members within the school on the day-to-day implementation of the phone policy. This may not be a true reflection of the time spent by these staff, although it closely correlated with self-reported time use from the teacher survey, which also indicated staff in restrictive schools spent less time overall on implementing their phone policy compared with permissive schools. Bootstrap analysis was used to address uncertainty around the mean time-use estimates provided by staff, although this does not address potential uncertainty in measurement error from staff time being provided by one staff member. This analysis demonstrated that restrictive phone policies were highly likely (76%) to be cost-saving. It also provides policy-makers with estimates of the likelihood of such policies being cost effective at different willingness-to-pay thresholds for QALYs and MWALYs.

Finally, we did not consider the time cost of a policy design because schools reported this to have taken place in school years prior to data collection. We also did not include the cost of phone pouches for storing phones on the school premises because, at the time of data collection, their use was rare among schools in the SMART Schools study wider sampling frame.^17^

In conclusion, this economic analysis of school smartphone policies suggests that school staff spend a large proportion of their time managing phone use in schools, regardless of phone policy type. Restrictive phone policies (compared with permissive policies) may be cost savings to schools in terms of reducing the staff time spent dealing with phone-related behaviours. In doing so, they may bring indirect benefits to schools and pupils by diverting staff time away from dealing with pupil phone use towards other more beneficial activities. Approaches that address adolescent phone and media use related to well-being remain an important focus, and schools require new policies and practices to reduce the time spent on managing phone use during the school day. More broadly, this work highlights the need to better understand the trade-offs and optimisation of staff inputs in schools to support students’ well-being. The findings also emphasise the importance of developing better methods to conduct and communicate health economics research for the education sector, recognising the different ways in which evidence is used to support resource allocation decisions.^30^ On smartphones, we currently lack an evidence-based best practice approach to addressing phone and social media use by adolescents. Therefore, all new approaches need to be accompanied by robust evaluation.

## Supplementary material

10.1136/bmjment-2025-301892 online supplemental file 1

## Data Availability

Data are available upon reasonable request.
