# Supplementary material for: Health economics analysis of restrictive school smartphone policies in secondary schools in England (SMART Schools)
Source: BMJ Ment Health. 2026 Feb 10;29(1):e301892. doi: 10.1136/bmjment-2025-301892 (PMC12911762; doi:10.1136/bmjment-2025-301892)
Supplement: online supplemental file 1 [file bmjment-29-1-s001.docx]

**Supplementary Materials**

**Table of Contents**

Supplementary Figures (p.2-3)

- Figure S1. Study Profile (p.2)
- Figure S2. Complete Case Sample (p.3)

Supplementary Tables (p.4-10)

- Table S1. Different Types of School Phone Policies within the Study Sample (p.4)
- Table S2. Characteristics of the Sampling Frame, Recruited Sample, and Economic Analysis Sample (p.5)
- Table S3. Salary Inputs (p.6)
- Table S4. Missing Time Use Data from Senior Leadership Team Survey (p.7)
- Table S5. T-test for Difference in Missingness in SLT School Time Use Data by School Phone Policy Group (p.8)
- Table S6. T-test for Difference in Missingness in SLT School Time Use Data by Propensity Score Tercile (p.8)
- Table S7. T-test for Difference in Pupil Outcomes Between Non-Missing and Missing SLT Time Use Data (p.8)
- Table S8. Teacher-Reported Weekly Time Spent Applying Sanctions and Monitoring Behaviour (p.9)
- Table S9. Multiple Imputation Analysis (p.10)
- Table S10. Salary Sensitivity Analysis (p.10)
- Table S11. Outliers Sensitivity Analysis (p.10).
- Table S12. Full Regression Results for Cost-Utility Analysis (p.11)

Resource Use Identification and Measurement (p.12-14)

- Senior Leadership Team Questionnaire (p.12-13)
- Teacher Survey Questionnaire (p.14)

Patient Reported Outcome Measures for Health Outcomes in Pupil Survey (p.15-18)

- Child Health Utility (CHU-9D) (p.15-16)
- The Warwick-Edinburgh Mental Well-being Scale (WEMWBS) (p.17-18)

CHEERS Checklist (p.19-21)

References (p.22)

**Figure S1. Study Profile**

**
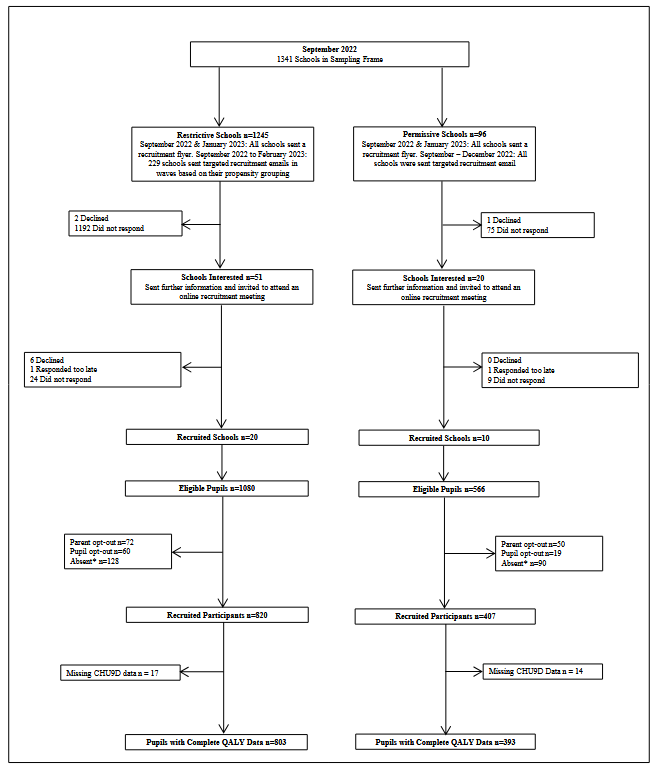
**

**Figure S2. Complete Case Sample**

**
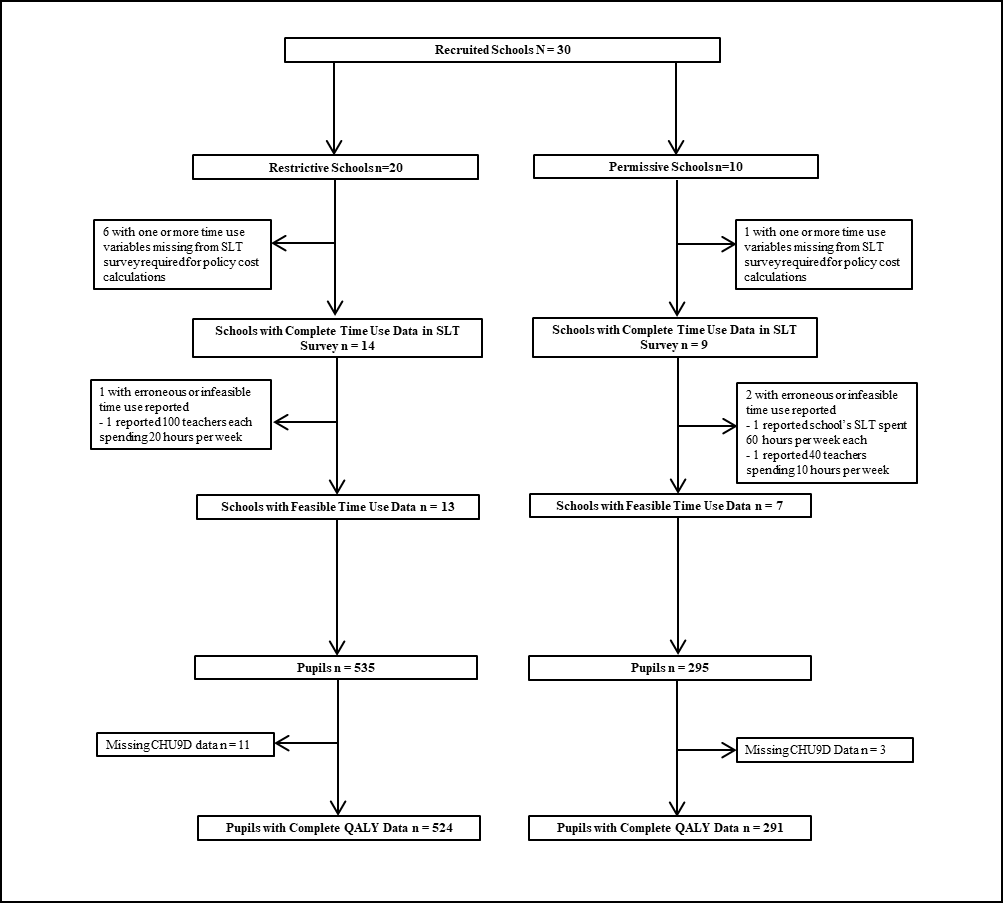
**

**Table S1. Different Types of School Phone Policies within the Study Sample**

| **Policy Categories** | **Number of Schools Adopting Policy Type** | **Sub-Categories** |
| --- | --- | --- |
| **Permissive Schools** | | |
| 1. Phones are allowed to be used at any time during the school day | 1 | N/A |
| 2. Phones are allowed to be used at school during certain times/in certain areas | 9 | (2a) Phones are allowed during lunch and break times (7 schools)  (2b) Phones are allowed to be used in designated zones (2 schools) |
| **Restrictive Schools** | | |
| 3. Phones are not allowed to be used at school but are accessible to pupils | 16 | Phones must be kept off inside bags (16 schools) |
| 4. Phones are not allowed to be used at school and are inaccessible to pupils | 4 | (4a) Phones must be kept in lockers (1 school)  (4b) Phones must be kept in a pouch (1 school)  (4c) Phones must be handed in to the school (1 school)  (4d) Phones are not allowed on school premises (1 school) |
| Source: Adapted from Goodyear VA, Randhawa A, Adab P et al.^3^ | | |

**Table S2. Characteristics of the Sampling Frame, Recruited Sample, and Economic Analysis Sample**

| Characteristics | All Schools | | | Restrictive Schools | | | Permissive Schools | | | Standardised Mean Difference  (Recruited Schools) |
| --- | --- | --- | --- | --- | --- | --- | --- | --- | --- | --- |
|  | Sampling Frame  (n=1,341) | Recruited  (n=30) | Economic Analysis  (n=22) | Sampling Frame  (n=1,245) | Recruited  (n=20) | Economic Analysis  (n=14) | Sampling Frame  (n=96) | Recruited  (n=10) | Economic Analysis  (n=8) | Restrictive vs Permissive |
| Academy Converter | 707 (52·72%) | 22 (73·33%) | 16 (72.73%) | 656 (52·69%) | 14 (70·00%) | 10 (71.43%) | 51 (53·13%) | 8 (80·00%) | 6 (75.00%) | -0.22 |
| Urban | 1153 (85·98%) | 25 (83·33%) | 18 (81.82%) | 1075 (86·34%) | 17 (85·00%) | 11 (78.57%) | 78 (81·25%) | 8 (80·00%) | 7 (87.50%) | 0.13 |
| Single Sex Schools | 130 (9·69%) | 3 (10·00%) | 3 (13.64%) | 117 (9·40%) | 1 (5·00%) | 1 (7.14%) | 13 (13·54%) | 2 (20·00%) | 2 (25.00%) | -0.44 |
| Pupils from Black or Minority Ethnic Groups % | 33·86% | 32·56% | 28.46% | 33·95% | 30·89% | 26.38% | 32·64% | 35·91% | (32.10%) | -0.18 |
| Pupils with EAL % | 16·92% | 15·87% | 12.59% | 16·92% | 15·67% | 13.71% | 16·92% | 16·29% | (13.76%) | -0.04 |
| Pupils Eligible for FSM % | 22·36% | 17·90% | 16.05% | 22·45% | 18·85% | 17.39% | 21·29% | 16·00% | (13.71%) | 0.30 |
| Pupils Eligible for SEN Support % | 12·50% | 11·80% | 11.30% | 12·50% | 13·00% | 12.07% | 12·00% | 10·00% | (9.95%) | 0.48 |
| In top 50% IDACI Decile | 668 (49·81%) | 16 (53·33%) | 12 (54.55%) | 619 (49·71%) | 10 (50·00%) | 8 (57.14%) | 49 (51·04%) | 6 (60·00%) | 4 (50.00%) | 0.19 |
| Sixth Form | 840 (62·64%) | 22 (73·33%) | 16 (72.73%) | 782 (62·81%) | 15 (75·00%) | 10 (71.43%) | 57 (59·34%) | 7 (70·00%) | 6 (75.00%) | 0.10 |
| Selective Admissions | 73 (5·44%) | 4 (13·33%) | 4 (18.18%) | 60 (4·82%) | 1 (5·00%) | 1 (7.14%) | 13 (13·54%) | 3 (30·00%) | 3 (37.5%) | -0.66 |
| Faith School | 259 (19·31%) | 5 (16·67%) | 2 (9.09%) | 246 (19·76%) | 2 (10·00%) | 0 (0.00%) | 13 (13·54%) | 3 (30·00%) | 2 (25.00%) | -0.49 |
| Figures reported as N (%) unless otherwise stated. Figures correct to 2 decimal places. *, **, *** indicates statistical significance at the 10%, 5%, and 1% level respectively. EAL = English as an Additional Language, FSM = Free School Meals, SEN = Special Education Needs, IDACI = Income Deprivation Affecting Children Index | | | | | | | | | | |

**Table S3. Salary Inputs**

| **Local Authority** | **Teaching Staff Mean Annual Salary** | **Teaching Staff Mean Per Hour Salary** | **Teaching Staff Median Annual Salary** | **Teaching Staff Median Per Hour Salary** | **SLT Mean Annual Salary** | **SLT Mean Per Hour Salary** | **SLT Median Annual Salary** | **SLT Median Per Hour Salary** | **Head Mean Annual Salary** | **Head Mean Per Hour Salary** | **Head Median Annual Salary** | **Head Median Per Hour Salary** |  |
| --- | --- | --- | --- | --- | --- | --- | --- | --- | --- | --- | --- | --- | --- |
| Bracknell Forest | 40,956 | 32.38 | 42,695 | 33.75 | 67,803 | 53.60 | 65,450 | 51.74 | 103,680 | 81.96 |  |  |  |
| Bristol, City Of | 40,136 | 31.73 | 41,604 | 32.89 | 63,371 | 50.10 | 61,041 | 48.25 | 93,917 | 74.24 | 94,898 | 75.02 |  |
| Buckinghamshire | 40,700 | 32.17 | 41,604 | 32.89 | 63,824 | 50.45 | 62,561 | 49.46 | 98,129 | 77.57 | 98,467 | 77.84 |  |
| Coventry | 40,013 | 31.63 | 41,522 | 32.82 | 61,902 | 48.93 | 61,042 | 48.25 | 93,372 | 73.81 | 91,502 | 72.33 |  |
| Gloucestershire | 40,203 | 31.78 | 41,604 | 32.89 | 60,500 | 47.83 | 59,557 | 47.08 | 95,293 | 75.33 | 96,763 | 76.49 |  |
| Leicestershire | 40,282 | 31.84 | 41,604 | 32.89 | 59,031 | 46.66 | 58,135 | 45.96 | 94,905 | 75.02 | 91,502 | 72.33 |  |
| Lincolnshire | 40,769 | 32.23 | 41,604 | 32.89 | 62,080 | 49.08 | 61,166 | 48.35 | 94,135 | 74.41 | 91,145 | 72.05 |  |
| Milton Keynes | 41,370 | 32.70 | 42,130 | 33.30 | 63,696 | 50.35 | 62,561 | 49.46 | 101,750 | 80.43 |  |  |  |
| North Northamptonshire | 39,360 | 31.11 | 40,490 | 32.01 | 61,996 | 49.01 | 59,579 | 47.10 | 95,095 | 75.17 | 97,272 | 76.89 |  |
| Nottinghamshire | 40,521 | 32.03 | 41,707 | 32.97 | 61,156 | 48.34 | 60,143 | 47.54 | 95,587 | 75.56 | 92,624 | 73.22 |  |
| Oxfordshire | 41,155 | 32.53 | 41,683 | 32.95 | 63,368 | 50.09 | 62,398 | 49.33 | 94,635 | 74.81 | 93,225 | 73.70 |  |
| Sandwell | 40,379 | 31.92 | 41,604 | 32.89 | 61,917 | 48.95 | 61,166 | 48.35 | 95,646 | 75.61 |  |  |  |
| Shropshire | 41,600 | 32.88 | 42,131 | 33.31 | 61,306 | 48.46 | 61,042 | 48.25 | 86,377 | 68.28 |  |  |  |
| Slough | 40,727 | 32.20 | 41,858 | 33.09 | 65,542 | 51.81 | 63,747 | 50.39 | 109,432 | 86.51 |  |  |  |
| Somerset | 40,727 | 32.19 | 42,131 | 33.31 | 59,002 | 46.64 | 58,135 | 45.96 | 88,016 | 69.58 | 86,050 | 68.02 |  |
| Staffordshire | 40,641 | 32.13 | 41,604 | 32.89 | 59,689 | 47.18 | 58,710 | 46.41 | 89,039 | 70.39 | 86,922 | 68.71 |  |
| Telford and Wrekin | 39,926 | 31.56 | 41,604 | 32.89 | 60,893 | 48.14 | 61,042 | 48.25 | 101,309 | 80.09 |  |  |  |
| Trafford | 40,764 | 32.22 | 41,604 | 32.89 | 63,000 | 49.80 | 61,166 | 48.35 | 96,528 | 76.31 |  |  |  |
| Warwickshire | 39,715 | 31.40 | 41,604 | 32.89 | 60,364 | 47.72 | 59,581 | 47.10 | 90,091 | 71.22 | 86,927 | 68.72 |  |
| Wirral | 41,911 | 33.13 | 43,416 | 34.32 | 64,710 | 51.15 | 62,561 | 49.46 | 105,234 | 83.19 |  |  |  |
| Worcestershire | 41,264 | 32.62 | 42,131 | 33.31 | 60,455 | 47.79 | 59,558 | 47.08 | 92,034 | 72.75 | 90,365 | 71.43 |  |
| Average (Sample) | 40,682 | 31.26 | 41,604 | 32.89 | 62,092 | 49.08 | 61,042 | 48.25 | 95,705 | 75.66 | 92,063 | 72.78 |  |
| Average (National) | 41,427 | 32.75 | 41,850 | 33.08 | 63,912 | 50.52 | 62,561 | 49.46 | 98,608 | 77.95 | 97,255 | 76.88 |  |
| Source: School Workforce in England 2022/23.^1^ Per hour salary based on a 1,265 hour working year.^2^ Teaching staff salary based on ‘Classroom Teacher’ data, SLT staff salary based on ‘Other Leadership Teacher’ data, Head salary based on ‘Headteacher Salary’. ‘Average’ refers to mean in the mean salaries columns and median in the median salaries columns. Blank entries indicate no data was available. | | | | | | | | | | | | | |

Salary estimates for all teaching staff were obtained from the School Workforce in England 2022/23.^18^ The local authority level mean annual salaries were used for ‘Headteacher’ (applied to headteacher group), ‘Other leadership teacher’ (applied to SLT group), and ‘Classroom teacher’ (applied to teaching staff group). To derive an estimated per hour salary, the annual salary was divided by 1,265 hours. Salary figures are presented in Table S2 (Supplementary Materials p·2).

Salary estimates for non-teaching staff were obtained from the National Joint Council (NJC) Local Government Pay Scales which are used to determine school support staff salaries.^19^ Spine point 7 was taken as a mid-point estimate of the salary (£22,369 per annum, £11·53 per hour) for all non-teaching staff, given that receptionists were the most significant contributor in hours in this group.

**Table S4. Missing Time Use Data from Senior Leadership Team Survey**

| **Time Use Variable** | **Missing: N (%)** |
| --- | --- |
| Academy Trust Board – Involved (Yes/No) | 0 (0%) |
| Academy Trust Board – Number Involved | 0 (0%) |
| Academy Trust Board – Typical Hours Per Week Per Individual | 0 (0%) |
| Headteacher – Involved (Yes/No) | 0 (0%) |
| Headteacher – N Involved | 5 (16.67%) |
| Headteacher – Typical Hours Per Week Per Individual | 6 (20.00%) |
| SLT – Involved (Yes/No) | 0 (0%) |
| SLT – N Involved | 3 (10.00%) |
| SLT – Typical Hours Per Week Per Individual | 5 (16.67%) |
| Teachers – Involved (Yes/No) | 0 (0%) |
| Teachers – N Involved | 5 (16.67%) |
| Teachers – Typical Hours Per Week Per Individual | 6 (20.00%) |
| Teaching Assistants – Involved (Yes/No) | 0 (0%) |
| Teaching Assistants – N Involved | 4 (13.33%) |
| Teaching Assistant – Typical Hours Per Week Per Individual | 6 (20.00%) |
| Safeguarding/Welfare Support – Involved (Yes/No) | 0 (0%) |
| Safeguarding/Welfare Support – N Involved | 4 (13.33%) |
| Safeguarding/Welfare Support – Typical Hours Per Week Per Individual | 5 (16.67%) |
| Receptionists – Involved (Yes/No) | 0 (0%) |
| Receptionists – N Involved | 2 (6.67%) |
| Receptionists – Typical Hours Per Week Per Individual | 3 (10.00%) |
| Caretakers – Involved (Yes/No) | 0 (0%) |
| Caretakers – N Involved | 2 (6.67%) |
| Caretakers – Typical Hours Per Week Per Individual | 2 (6.67%) |
| Cleaners – Involved (Yes/No) | 0 (0%) |
| Cleaners – N Involved | 2 (6.67%) |
| Cleaners – Typical Hours Per Week Per Individual | 2 (6.67%) |
| Other – Involved (Yes/No) | 0 (0%) |
| Other – Specify | 2 (6.67%) |
| Other – Typical Hours Per Week Per Individual | 0 (0%) |
| Figures correct to 2 decimal places | |

**Table S5. T-test for Difference in Missingness in SLT School Time Use Data by School Phone Policy Group**

| **Group** | **N** | **Missing - Mean**  **(S.D.)** | **Difference**  **(p-value)** |
| --- | --- | --- | --- |
| Restrictive | 20 | 0.350  (0.489) | 0.050  (0.793) |
| Permissive | 10 | 0.300  (0.483) |  |
| Statistical significance indicated by *, **, and *** at the 1%, 5%, and 10% level respectively. P-value obtained from testing the null hypothesis of difference in means equals 0 using a two-sided t-test. | | | |

**Table S6. T-test for Difference in Missingness in SLT School Time Use Data by Propensity Score Tercile**

| **Propensity Score Tercile** | **N** | **Missing – Mean**  **(S.D)** | **Difference: 1 vs 2**  **(p-value)** | **Difference: 2 vs 3**  **(p-value)** | **Difference: 1 vs 3**  **(p-value)** |
| --- | --- | --- | --- | --- | --- |
| 1 | 10 | 0.400  (0.516) | -0.029  (0.913) | -0.029  (0.384) | 0.169  (0.405) |
| 2 | 7 | 0.428  (0.535) |  |  |  |
| 3 | 13 | 0.231  (0.470) |  |  |  |
| Statistical significance indicated by *, **, and *** at the 1%, 5%, and 10% level respectively. P-value obtained from testing the null hypothesis of difference in means equals 0 using a two-sided t-test. | | | | | |

**Table S7. T-test for Difference in Pupil Outcomes Between Non-Missing and Missing Time SLT Time Use Data**

| **Variable** | **N** | **Mean:**  **Non-Missing Time Use Data**  **(S.D.)** | **Mean:**  **Missing Time Use Data**  **(S.D.)** | **Difference**  **(p-value)** |
| --- | --- | --- | --- | --- |
| QALYs | 1,196 | 0.604  (0.089) | 0.613  (0.090) | -0.008  (0.126) |
| MWALYs | 1,211 | 0.518  (0.136) | 0.523  (0.137) | -0.005  (0.561) |
| Statistical significance indicated by *, **, and *** at the 1%, 5%, and 10% level respectively. P-value obtained from testing the null hypothesis of difference in means equals 0 using a two-sided t-test. | | | | |

**Table S8. Teacher-Reported Weekly Time in Hours Spent Applying Sanctions and Monitoring Behaviour**

| **Activity (hours)** | **Restrictive**  **(N = 21)** | **Permissive**  **(N = 15)** | **Difference** |
| --- | --- | --- | --- |
|  | **Mean**  **(S.D.)** | **Mean**  **(S.D.)** | **Mean**  **(p-value)** |
| Monitoring students’ phone use at break and/or lunchtimes | 0.725  (0.976) | 0.538  (0.721) | 0.187  (0.559) |
| Verbal warning/reprimanding pupil phone use | 0.696  (1.011) | 0.707  (0.884) | -0.012  (0.972) |
| Recording incidents (e.g., in planner or on school system) | 0.800  (2.210) | 2.677  (8.222) | -1.876  (0.326) |
| Organising and leading detentions | 0.624  (0.782) | 0.315  (0.476) | 0.308  (0.210) |
| Managing the confiscation of phones | 0.205  (0.317) | 0.454  (0.621) | -0.249  (0.132) |
| Managing isolation room for pupil behaviour (or equivalent) | 0.452  (0.865) | 0.423  (1.382) | 0.029  (0.940) |
| 1:1 meetings with pupils | 0.981  (1.790) | 0.907  (1.635) | 0.073  (0.905) |
| Sending text, letter, or email to parents | 1.000  (1.508) | 0.700  (1.142) | 0.300  (0.543) |
| Discussion with parents (by phone or in school) | 1.490  (2.568) | 0.815  (1.674) | 0.675  (0.407) |
| Providing staff with information on the policy | 0.029  (0.110) | 0.423  (1.382) | -0.395  (0.198) |
| Providing staff with information on behaviour incidents related to phone use | 0.255  (0.679) | 0.496  (1.381) | -0.241  (0.500) |
| Staff training relating to the phone policy | 0.012  (0.055) | 0.076  (0.277) | -0.065  (0.301) |
| Other (specify) 1 | 0.048  (0.218) | 0.239  (0.597) | -0.191  (0.190) |
| Other (specify) 2 | 0.000  (0.000) | 0.308  (1.109) | -0.308  (0.209) |
| Total | 7.282  (10.469) | 9.081  (14.006) | -1.798  (0.672) |
| Total Sanctions | 5.448  (7.734) | 4.323  (6.721) | 1.125  (0.668) |
| Total Administration | 1.786  (3.368) | 4.212  (8.757) | -2.425  (0.260) |
| Statistical significance indicated by *, **, and *** at the 0.1%, 1%, and 5% level respectively. P-value obtained from testing the null hypothesis of difference in means equals 0 using a two-sided t-test. ‘Sanctions’ variables are: Verbal warning/reprimanding pupil phone use, Organising and leading detentions, Managing the confiscation of phones, Managing isolation room for pupil behaviour (or equivalent), 1:1 meetings with pupils, Sending text, letter, or email to parents, Discussion with parents (by phone or in school). ‘Administration’ variables are: Monitoring students’ phone use at break and/or lunchtimes, Recording incidents (e.g., in planner or on school system), Providing staff with information on the policy, Providing staff with information on behaviour incidents related to phone use, Staff training relating to the phone policy. | | | |

**Table S9. Multiple Imputation Analysis**

| **Model** | **Average Coefficient**  **(S.E)** | **95% CI** | **ICER** |
| --- | --- | --- | --- |
| School Year Total Cost Per Pupil | -174.800  (109.477) | -389.371 to 39.771 | - |
| QALYs | -0.005  (0.008) | -0.020 to 0.009 | 34,960 |
| MWALYs | -0.009  (0.012) | -0.032 to 0.014 | 19,422 |
| Estimates obtained from 30 imputed datasets, combined using Rubin’s rule.^4^ | | | |

**Table S10. Salary Sensitivity Analysis**

| **Dependent Variable** | **Local Authority Median:**  **Estimated Difference**  **(95% CI)** | **Sample Average Mean:**  **Estimated Difference**  **(95% CI)** | **Sample Average Median:**  **Estimated Difference**  **(95% CI)** | **National Average**  **Mean:**  **Estimated Difference**  **(95% CI)** | **National Average**  **Median:**  **Estimated Difference**  **(95% CI)** |
| --- | --- | --- | --- | --- | --- |
| School Year Total Cost Per Pupil (£) | -96.22  (-232.93 – 40.49) | -94.48  (-220.62 – 31.66) | -98.62  (-229.81 – 32.57) | -98.77  (-230.52 – 32.98) | -99.38  (-231.77 – 33.01) |
| 95% confidence intervals in parentheses. Statistical significance indicated by *, **, and *** at the 1%, 5%, and 10% level respectively. | | | | | |

**Table S11. Outliers Sensitivity Analysis**

| **Cost and Outcomes** | **Restrictive:**  **Mean**  **(95% CI)** | **Permissive:**  **Mean**  **(95% CI)** | **Difference:**  **Mean**  **(95% CI)** |  |
| --- | --- | --- | --- | --- |
| School Year Total Cost Per Pupil (£) | 139.61  (8.22 – 270.99) | 490.44  (48.04 – 932.84) | -350.83  (-819.35 – 117.69) |  |
| QALYs | 0.608  (0.598 – 0.618) | 0.596  (0.581 – 0.611) | 0.012  (-0.009 – 0.034) |  |
| MWALYs | 0.518  (0.501 – 0.535) | 0.520  (0.494 – 0.546) | -0.002  (-0.039 – 0.035) |  |
| 95% confidence intervals in parentheses. Statistical significance indicated by *, **, and *** at the 0.1%, 1%, and 5% level respectively. Figures correct to three decimal places (outcomes) or two decimal places (cost). | | | | |

**Table S12. Full Regression Results for Cost-Utility Analysis**

| **Variable** | **Cost Per Pupil** | **QALYs** | **MWALYs** |
| --- | --- | --- | --- |
|  | Coefficient  (95% CI) | Coefficient  (95% CI) | Coefficient  (95% CI) |
| **School Level** | | | |
| Restrictive Policy | -94.05  (-229.25 – 41.14) | 0.009  (-0.014 – 0.032) | -0.004  (-0.044 – 0.036) |
| IDACI Decile | 14.34  (-23.12 – 51.80) | 0.003  (-0.003 – 0.008) | 0.000  (-0.009 – 0.009) |
| Roll Size | 0.08  (-0.16 – 0.32) | -0.000  (-0.000 – 0.000) | 0.000  (-0.000 – 0.000) |
| Secular School | 126.18  (-192.12 – 444.48) | -0.049**  (-0.095 – 0.002) | -0.047  (-0.127 – 0.033) |
| Non-Selective School | -234.28  (-705.67 – 238.11) | 0.022  (-0.047 – 0.091) | 0.018  (-0.102 – 0.137) |
| Single-Sex School | 42.66  (-77.56 – 157.87) | -0.008  (-0.053 – 0.036) | 0.004  (-0.073 – 0.080) |
| Percentage SEN Pupils | 156.09  (-1,131.96 – 1,444.13) | 0.087  (-0.190 – 0.364) | -0.134  (-0.610 – 0.342) |
| Percentage EAL Pupils | -7.66**  (-15.03 – -0.283) | 0.002**  (0.000 – 0.003) | 0.000  (-0.001 – 0.003) |
| Percentage FSM Pupils | 23.46**  (0.30 – 46.63) | -0.003*  (-0.007 – 0.000) | -0.003  (-0.009 – 0.004) |
| **Pupil Level** | | | |
| Gender:  Male | - | 0.042***  (0.029 – 0.055) | 0.066***  (0.047 – 0.086) |
| Gender:  Prefer Not to Say | - | -0.052  (-0.117 – 0.013) | -0.091*  (-0.183 – 0.001) |
| Ethnicity:  Black, African, Caribbean, Black British | - | -0.011  (-0.049 – 0.026) | 0.001  (-0.057 – 0.059) |
| Ethnicity:  Mixed, Multiple Ethnic Groups | - | -0.025  (-0.053 – 0.004) | -0.035  (-0.079 – 0.008) |
| Ethnicity:  Other, Prefer Not to Say | - | -0.007  (-0.044 – 0.028) | -0.007  (-0.059 – 0.046) |
| Ethnicity:  White | - | -0.018*  (-0.036 – 0.003) | 0.011  (-0.017 – 0.038) |
| Season of Response:  Summer | - | 0.008  (-0.013 – 0.029) | -0.000  (-0.037 – 0.035) |
| **ICC** | | | |
| School | - | 0.000  (0.000 – 0.000) | 0.011  (0.000 – 0.163) |
| Year 10 within School | - | 0.009  (0.000 – 0.166) | 0.017  (0.003 – 0.096) |
| Notes: . *, **, *** indicates statistical significance at the 10%, 5%, and 1% level respectively. EAL = English as an Additional Language, FSM = Free School Meals, SEN = Special Education Needs, IDACI = Income Deprivation Affecting Children Index. Intraclass Correlation Coefficient (ICC) represents the proportion of residual variance in the outcome attributable to clustering at each level of the multilevel model. | | | |

**Resource Use Identification and Measurement**

**Senior Leadership Team Questionnaire**

Question identifying individuals

Which individuals are involved on a day-to-day basis implementing the school’s phone policy (e.g., (e.g. monitoring behaviour, recording incidents, duty roles, detentions, communicating with parents, talking to pupils, managing phones, supporting other school sta, providing training to other staff) (please select all that apply)

1. Academy Trust Board (or equivalent)
2. Headteacher
3. Senior Leadership Team
4. School Governors
5. Teachers
6. Teaching Assistants
7. Safeguarding/Welfare Support Staff
8. School Reception Staff
9. Building Manager/Caretakers
10. Cleaners
11. Other (please specify)

Questions identifying resource use

Academy Trust Board (or equivalent)

i) How many of those individuals are involved in implementing the school’s phone policy?

ii) How many hours does a typical member of this staff spend per week on tasks related to the school’s phone policy (e.g., monitoring behaviour, recording incidents, duty toles, detentions, communicating with parents, talking to pupils, managing phones, supporting other school staff, providing training to other staff)

Headteacher

i) How many of those individuals are involved in implementing the school’s phone policy?

ii) How many hours does a typical member of this staff spend per week on tasks related to the school’s phone policy (e.g., monitoring behaviour, recording incidents, duty toles, detentions, communicating with parents, talking to pupils, managing phones, supporting other school staff, providing training to other staff)

Senior Leadership Team

i) How many of those individuals are involved in implementing the school’s phone policy?

ii) How many hours does a typical member of this staff spend per week on tasks related to the school’s phone policy (e.g., monitoring behaviour, recording incidents, duty toles, detentions, communicating with parents, talking to pupils, managing phones, supporting other school staff, providing training to other staff)

School Governors

i) How many of those individuals are involved in implementing the school’s phone policy?

ii) How many hours does a typical member of this staff spend per week on tasks related to the school’s phone policy (e.g., monitoring behaviour, recording incidents, duty toles, detentions, communicating with parents, talking to pupils, managing phones, supporting other school staff, providing training to other staff)

Teachers

i) How many of those individuals are involved in implementing the school’s phone policy?

ii) How many hours does a typical member of this staff spend per week on tasks related to the school’s phone policy (e.g., monitoring behaviour, recording incidents, duty toles, detentions, communicating with parents, talking to pupils, managing phones, supporting other school staff, providing training to other staff)

Teaching Assistants

i) How many of those individuals are involved in implementing the school’s phone policy?

ii) How many hours does a typical member of this staff spend per week on tasks related to the school’s phone policy (e.g., monitoring behaviour, recording incidents, duty toles, detentions, communicating with parents, talking to pupils, managing phones, supporting other school staff, providing training to other staff)

Safeguarding/Welfare Support Staff

i) How many of those individuals are involved in implementing the school’s phone policy?

ii) How many hours does a typical member of this staff spend per week on tasks related to the school’s phone policy (e.g., monitoring behaviour, recording incidents, duty toles, detentions, communicating with parents, talking to pupils, managing phones, supporting other school staff, providing training to other staff)

School Reception Staff

i) How many of those individuals are involved in implementing the school’s phone policy?

ii) How many hours does a typical member of this staff spend per week on tasks related to the school’s phone policy (e.g., monitoring behaviour, recording incidents, duty toles, detentions, communicating with parents, talking to pupils, managing phones, supporting other school staff, providing training to other staff)

Building Managers/Caretakers

i) How many of those individuals are involved in implementing the school’s phone policy?

ii) How many hours does a typical member of this staff spend per week on tasks related to the school’s phone policy (e.g., monitoring behaviour, recording incidents, duty toles, detentions, communicating with parents, talking to pupils, managing phones, supporting other school staff, providing training to other staff)

Cleaners

i) How many of those individuals are involved in implementing the school’s phone policy?

ii) How many hours does a typical member of this staff spend per week on tasks related to the school’s phone policy (e.g., monitoring behaviour, recording incidents, duty toles, detentions, communicating with parents, talking to pupils, managing phones, supporting other school staff, providing training to other staff)

Others

i) If you selected ‘other’ please specify here

ii) How many hours does a typical member of this staff spend per week on tasks related to the school’s phone policy (e.g., monitoring behaviour, recording incidents, duty toles, detentions, communicating with parents, talking to pupils, managing phones, supporting other school staff, providing training to other staff)

**Teacher Survey Questionnaire**

How much time, in a typical week, do you spend on the following tasks related to the school’s phone policy:

1. Monitoring students’ phone use at break and/or lunchtime (e.g., when on duty)
2. Verbal warning/reprimanding pupil phone use
3. Recording incidents (e.g., in planner or on school system)
4. Organising and leading detentions
5. Managing the confiscation of phones
6. Managing isolation room for pupil behaviour (or equivalent)
7. 1:1 meetings with pupil
8. Sending text, letter, or email to parents
9. Discussion with parents (by phone or in school)
10. Supporting other school staff
11. Providing staff with information on the policy
12. Providing staff with information on behaviour incidents related to phone use
13. Staff training relating to the phone policy

Are there any other tasks during the school day related to the school’s phone policy that you engage with, and how much time do you spend on these?

1. Other (please specify)
   1. How many hours?
2. Other (please specify)
   1. How many hours?
3. Other (please specify)
   1. How many hours?

**Patient Reported Outcome Measures for Health Outcomes in Pupil Survey**

**Child Health Utility (CHU-9D)^4^**

“This section asks you questions about your feelings and what we call “quality of life”. These following questions asks about how you are today. For each question, please read all the choices and decided which one is most like you today. Select only one box for each question.”

**1. Worried**

- I don’t feel worried today
- I feel a little bit worried today
- I feel a bit worried today
- I feel quite worried today
- I feel very worried today

**2. Sad**

- I don’t feel sad today
- I feel a little bit sad today
- I feel a bit sad today
- I feel quite sad today
- I feel very sad today

**3. Pain**

- I don’t have any pain today
- I have a little bit of pain today
- I have a bit of pain today
- I have a lot of pain today

**4. Tired**

- I don’t feel tired today
- I feel a little bit tired today
- I feel a bit tired today
- I feel quite tired today
- I feel very tired today

**5. Annoyed**

- I don’t feel annoyed today
- I feel a little bit annoyed today
- I feel a bit annoyed today
- I feel quite annoyed today
- I feel very annoyed today

**6. School Work/Homework (such as reading, writing, doing lessons)**

- I have no problems with my schoolwork/homework today
- I have a few problems with my schoolwork/homework today
- I have some problems with my schoolwork/homework today
- I have many problems with my schoolwork/homework today
- I can’t do my schoolwork/homework today

**7. Sleep**

- Last night I had no problems sleeping
- Last night I had a few problems sleeping
- Last night I had some problems sleeping
- Last night I had many problems sleeping
- Last night I couldn’t sleep at all

**8. Daily routine (things like eating, having a bath/shower, getting dressed)**

- I have no problems with my daily routine today
- I have a few problems with my daily routine today
- I have some problems with my daily routine today
- I have many problems with my daily routine today
- I can’t do my daily routine today

**9. Able to join in activities (things like playing our with your friends, doing sports, joining in things)**

- I can join in with any activities today
- I can join in with most activities today
- I can join in with some activities today
- I can join in with a few activities today
- I can join in with no activities today

**The Warwick-Edinburgh Mental Well-being Scale (WEMWBS)^5,6^**

“This section asks you some questions about your mental wellbeing. Below are some statements about feelings and thoughts. Please tick to select which option best describes your experience of each over the last 2 weeks (please select only one option for each statement)”

“I’ve been feeling optimistic about the future” *****

- None of the time
- Rarely
- Some of the time
- Often
- All of the time

“I’ve been feeling useful” *****

- None of the time
- Rarely
- Some of the time
- Often
- All of the time

“I’ve been feeling relaxed” *****

- None of the time
- Rarely
- Some of the time
- Often
- All of the time

“I’ve been feeling interested in other people”

- None of the time
- Rarely
- Some of the time
- Often
- All of the time

“I’ve had energy to spare”

- None of the time
- Rarely
- Some of the time
- Often
- All of the time

“I’ve been dealing with problems well” *****

- None of the time
- Rarely
- Some of the time
- Often
- All of the time

“I’ve been thinking clearly” *****

- None of the time
- Rarely
- Some of the time
- Often
- All of the time

“I’ve been feeling good about myself”

- None of the time
- Rarely
- Some of the time
- Often
- All of the time

“I’ve been feeling close to other people” *****

- None of the time
- Rarely
- Some of the time
- Often
- All of the time

“I’ve been feeling confident”

- None of the time
- Rarely
- Some of the time
- Often
- All of the time

“I’ve been able to make up my own mind about things” *****

- None of the time
- Rarely
- Some of the time
- Often
- All of the time

“I’ve been feeling loved”

- None of the time
- Rarely
- Some of the time
- Often
- All of the time

“I’ve been interested in new things”

- None of the time
- Rarely
- Some of the time
- Often
- All of the time

“I’ve been feeling cheerful”

- None of the time
- Rarely
- Some of the time
- Often
- All of the time

***** indicates items in the SWEMWBS used for calculating MWALYs

**CHEERS Checklist^8^**

# CHEERS 2022 Checklist

| **Topic** | **No.** | **Item** | **Location where item is reported** |
| --- | --- | --- | --- |
| **Title** |  |  |  |
|  | 1 | Identify the study as an economic evaluation and specify the interventions being compared. | Objective (p5-6) |
| **Abstract** |  |  |  |
|  | 2 | Provide a structured summary that highlights context, key methods, results, and alternative analyses. | Abstract (p2), Key Messages (p3) |
| **Introduction** |  |  |  |
| **Background and objectives** | 3 | Give the context for the study, the study question, and its practical relevance for decision making in policy or practice. | Background (p4-6) |
| **Methods** |  |  |  |
| **Health economic analysis plan** | 4 | Indicate whether a health economic analysis plan was developed and where available. | Not reported - protocol published and available |
| **Study population** | 5 | Describe characteristics of the study population (such as age range, demographics, socioeconomic, or clinical characteristics). | Methods (p6-7); Table 1 (p13) |
| **Setting and location** | 6 | Provide relevant contextual information that may influence findings. | Methods (p6-7) |
| **Comparators** | 7 | Describe the interventions or strategies being compared and why chosen. | Methods (p6), Table S1 |
| **Perspective** | 8 | State the perspective(s) adopted by the study and why chosen. | Methods (p7) |
| **Time horizon** | 9 | State the time horizon for the study and why appropriate. | Methods (p7) |
| **Discount rate** | 10 | Report the discount rate(s) and reason chosen. | Not Applicable |
| **Selection of outcomes** | 11 | Describe what outcomes were used as the measure(s) of benefit(s) and harm(s). | Methods (p9) |
| **Measurement of outcomes** | 12 | Describe how outcomes used to capture benefit(s) and harm(s) were measured. | Methods (p9) |
| **Valuation of outcomes** | 13 | Describe the population and methods used to measure and value outcomes. | Methods (p9) |
| **Measurement and valuation of resources and costs** | 14 | Describe how costs were valued. | Methods (p8) |
| **Currency, price date, and conversion** | 15 | Report the dates of the estimated resource quantities and unit costs, plus the currency and year of conversion. | Methods (p7-8) |
| **Rationale and description of model** | 16 | If modelling is used, describe in detail and why used. Report if the model is publicly available and where it can be accessed. | Not Applicable |
| **Analytics and assumptions** | 17 | Describe any methods for analysing or statistically transforming data, any extrapolation methods, and approaches for validating any model used. | Not Applicable |
| **Characterising heterogeneity** | 18 | Describe any methods used for estimating how the results of the study vary for subgroups. | Not Applicable |
| **Characterising distributional effects** | 19 | Describe how impacts are distributed across different individuals or adjustments made to reflect priority populations. | Not Applicable |
| **Characterising uncertainty** | 20 | Describe methods to characterise any sources of uncertainty in the analysis. | Methods (p10-11) |
| **Approach to engagement with patients and others affected by the study** | 21 | Describe any approaches to engage patients or service recipients, the general public, communities, or stakeholders (such as clinicians or payers) in the design of the study. | Methods (p7) |
| **Results** |  |  |  |
| **Study parameters** | 22 | Report all analytic inputs (such as values, ranges, references) including uncertainty or distributional assumptions. | Results (p11-13); Table 1 (p13); Table S3, Table S8 |
| **Summary of main results** | 23 | Report the mean values for the main categories of costs and outcomes of interest and summarise them in the most appropriate overall measure. | Results (p14); Table 2 (p14) |
| **Effect of uncertainty** | 24 | Describe how uncertainty about analytic judgments, inputs, or projections affect findings. Report the effect of choice of discount rate and time horizon, if applicable. | Results (p14-16); Figure 1a-b; Figure 2; Tables S9-10 |
| **Effect of engagement with patients and others affected by the study** | 25 | Report on any difference patient/service recipient, general public, community, or stakeholder involvement made to the approach or findings of the study | Not Applicable |
| **Discussion** |  |  |  |
| **Study findings, limitations, generalisability, and current knowledge** | 26 | Report key findings, limitations, ethical or equity considerations not captured, and how these could affect patients, policy, or practice. | Discussion (p16-19) |
| **Other relevant information** |  |  |  |
| **Source of funding** | 27 | Describe how the study was funded and any role of the funder in the identification, design, conduct, and reporting of the analysis | Declarations (p19) |
| **Conflicts of interest** | 28 | Report authors conflicts of interest according to journal or International Committee of Medical Journal Editors requirements. | ICJME and COI Statements Provided |

*From:* Husereau D, Drummond M, Augustovski F, et al. Consolidated Health Economic Evaluation Reporting Standards 2022 (CHEERS 2022) Explanation and Elaboration: A Report of the ISPOR CHEERS II Good Practices Task Force. Value Health 2022;25. <doi:10.1016/j.jval.2021.10.008>

**References**

1. Department for Education. School Workforce in England 2022/23. Online. Available from: <https://explore-education-statistics.service.gov.uk/find-statistics/school-workforce-in-england>
2. Department for Education. School teachers’ pay and conditions document 2022 and guidance on school teachers’ pay and conditions. Online. Available from: <https://assets.publishing.service.gov.uk/media/634839ea8fa8f534627a6e15/2022_STPCD.pdf>
3. Goodyear VA, Randhawa A, Adab P et al. School phone policies and their association with mental wellbeing, phone use and social media use (SMART Schools): a cross-sectional observational study. *The Lancet Regional Health Europe*. 2025; Online first <https://doi.org/10.1016/j.lanepe.2025.101211>
4. Rubin, DB. Estimating causal effects from large data sets using propensity scores. *Ann Intern Med* 1997; **127(8 Pt 2)**: 757-763.
5. Stevens S. Developing a descriptive system for a new preference-based measure of health-related quality of life for children. *Qual Life Res* 2009; **18(8):** 1105–1113
6. Tennant R., Hiller L, Fishwick R et al. The Warwick-Edinburgh Mental Well-being Scale (WEMWBS): development and UK validation. *Health Qual Life Outcomes* 2007; **5:** 63
7. Yiu HHE, Buckell J, Petrou S, Stewart-Brown S, Madan J. Derivation of a UK preference-based value set for the Short Warwick-Edinburgh Mental Well-being Scale (SWEMWBS) to allow estimation of Mental Well-being Adjusted Life Years (MWALYs). *Social Science & Medicine* 2023; 115928
8. Husereau D, Drummond M, Augustovski F, et al. Consolidated Health Economic Evaluation Reporting Standards 2022 (CHEERS 2022) Explanation and Elaboration: A Report of the ISPOR CHEERS II Good Practices Task Force. *Value Health 2022*;**25**. <doi:10.1016/j.jval.2021.10.008>
